# Information Theory and Cognition: A Review

**DOI:** 10.3390/e20090706

**Published:** 2018-09-14

**Authors:** Khalid Sayood

**Affiliations:** Department of Electrical and Computer Engineering, University of Nebraska, Lincoln, NE 68588-0511, USA; ksayood@unl.edu; Tel.: +1-402-472-6688

**Keywords:** information theory, cognition, neuronal codes, average mutual information, predictive coding

## Abstract

We examine how information theory has been used to study cognition over the last seven decades. After an initial burst of activity in the 1950s, the backlash that followed stopped most work in this area. The last couple of decades has seen both a revival of interest, and a more firmly grounded, experimentally justified use of information theory. We can view cognition as the process of transforming perceptions into information—where we use information in the colloquial sense of the word. This last clarification is one of the problems we run into when trying to use information theoretic principles to understand or analyze cognition. Information theory is mathematical, while cognition is a subjective phenomenon. It is relatively easy to discern a subjective connection between cognition and information; it is a different matter altogether to apply the rigor of information theory to the process of cognition. In this paper, we will look at the many ways in which people have tried to alleviate this problem. These approaches range from narrowing the focus to only quantifiable aspects of cognition or borrowing conceptual machinery from information theory to address issues of cognition. We describe applications of information theory across a range of cognition research, from neural coding to cognitive control and predictive coding.

## 1. Introduction

The connection between cognition and information theory seems obvious—both deal with information. However, using the tools of information theory to analyze cognition has been a difficult task. As pointed out by Shannon in his famous Bandwagon editorial [1], “the hard core of information theory is, essentially, a branch of mathematics, a strictly deductive system," while induction is an important aspect of science. Information theory uses a quantitative approach while analyses of cognition tend to be qualitative. The focus of information theory is on ensembles of messages while the focus of cognition is on a particular message rather than the ensemble. Shannon was not alone in his discomfort at the arbitrary application of information theory. Cronbach [2] argues strongly against the indiscriminate application of information theory to topics in psychology. Though some of his objections may be due to a misunderstanding of information theory, much of what he said was justifiably cautionary. While the subject of both information theory and cognition is information, the different ways of conceptualizing information has made the application of information theory to cognition difficult. The situation has changed in the last decade. A more systematic understanding of cognition and some very ingenious formulations of the cognition process has allowed the development of a relationship between information theory and the analysis and understanding of cognition.

The justification for the use of information theory in examining cognitive processes is the ability of the theory to provide a coherent understanding of experimental results. To apply information theory indiscriminately to any process that does not follow the standard communication theory paradigm would be wrong. However, the justification for the application of an investigative tool is based on the type of questions that can be asked. We plan to show in this survey of work on cognition and information theory that there are certain types of questions for which information theory is an excellent tool.

The first difficulty we face when we talk about cognition is definitional. What exactly is cognition? The Oxford English Dictionary defines cognitions as “The mental action or process of acquiring knowledge and understanding through thought, experience, and the senses.” This is a rather broad expanse of knowledge and our goal is in no way to provide a survey of all the work that has been done in the area of cognition. Instead, we will use the lens of information theory and examine how this particular perspective has impacted the study of cognition. We begin with a study of how information is transmitted over a single neuron. The stimulus-response behavior of a single neuron may seem very distant from the complex tasks we associate with the word cognition; however, single neurons can show surprisingly complex behavior [3,4,5] and can perhaps guide our understanding of more complex tasks. We will find that even focusing down to this level does not result in the clarity one would expect. On the other hand, this area, focusing mainly on neural coding, is the most quantitative aspect of cognition, making it the most natural entry point for the exploration of cognition with the quantitative tools of information theory. We begin with single neurons and the possible neural codes and proceed to signaling over multiple neurons using population codes.

We then turn to some behavioral results—some of them from the nineteenth century—that provide a strong justification for the use of information theory in the study of cognition. We will begin with the simplest results which show that the processing time and its correlates for stimuli vary with the self information of the event. We then go to the next step and show that response times for tasks involving selection from an alphabet vary with the entropy of the stimulus. We will examine the subject of cognitive control which has seen a great deal of attention since the development of various neuroimaging tools, in particular, functional Magnetic Resonance Imaging (fMRI). Moving to the more abstract, we look at model-based cognition. We then review the literature on predictive coding in cognition to explain cognitive processes. This is one of the most fertile areas of application of concepts from information theory and one which is becoming a very active area of research. In the context of predictive coding, we will describe our current understanding of the structures responsible for visual cognition—a clear example of form following function. We end with a discussion about cognitive load which in some sense ties all the various ideas together.

The applications of information theoretic concepts to various aspects of cognition have expanded so rapidly that it would be difficult to do justice to all topics. Therefore, there are a number of topics we will not cover. One is *integrated information theory* (*iit*) [6,7] which attempts to use the concept of information to deduce the neural substrates of consciousness—*iit* is not about cognition and it is not clear how much it has to do with information theory [8]. We will also not discuss the ways in which information theory can be used in constructing and analyzing stimulus sets or to Structural Information Theory. We refer those interested in this topic to *The processing of information and structure* [9,10]. We will also not discuss the relationship of self information or *surprisal* to linguistic structures [11,12,13,14]. Finally, we will not discuss the work on information decomposition and the application of multivariate information theory to the dynamics of neural ensembles. For these topics, we would refer interested readers to the work of Wibral et al. [15,16] and the special issues of *Entropy* [17], and of the *Journal of Computational Neuroscience* [18].

## 2. Information Theory—A Very Brief Introduction

We will begin with a brief introduction to some of the information theoretic quantities we will be referring to on this survey. As the same quantities are sometimes given different names, this will also serve to provide us with a common vocabulary.

If *X* is a discrete random variable that takes on values $\left\{x_{1},x_{2},\ldots ,x_{N}\right\}$ and $p_{X}\left(x_{i}\right)$ is the probability that the random variable *X* takes on the value $x_{i}$, then $i(x_{i})$, the *self-information* associated with the event that the random variable *X* takes on the value $x_{i}$, is given by

$$i\left(x_{i}\right)=\log\frac{1}{p_{X}\left(x_{i}\right)}=-\log p_{X}\left(x_{i}\right).$$

This intuitively makes sense: If the probability of the event is close to one, there is very little information associated with its occurrence, while the occurrence of an event of low probability has more information associated with it. This definition also makes sense if we consider the joint occurrence of two independent events. Given random variables *X* and *Y*, we can define the self information associated with the occurrence of the pair $\left(x_{i},y_{j}\right)$ as

$$i\left(x_{i},y_{j}\right)=-\log p_{XY}\left(x_{i},y_{j}\right).$$

If *X* and *Y* are independent, $p_{X,Y}\left(x_{i},y_{j}\right)=p_{X}\left(x_{i}\right)p_{Y}\left(y_{j}\right)$ and

$$i\left(x_{i},y_{j}\right)=-\log p_{X}\left(x_{i}\right)p_{Y}\left(y_{j}\right)=-\log p_{X}\left(x_{i}\right)-\log p_{Y}\left(y_{j}\right)=i\left(x_{i}\right)+i\left(y_{j}\right).$$

Thus, the self information associated with independent events is additive. Self information has also been called *Shannon information content* [19] and *surprisal* [20].

The expected value of the self-information is called the *entropy* associated with the random variable:

$$H\left(X\right)=E\left[i(x_{i})\right]=\sum_{i}i\left(x_{i}\right)p_{X}\left(x_{i}\right)=\sum_{i}p_{X}\left(x_{i}\right)\log\frac{1}{p_{X}\left(x_{i}\right)}=-\sum_{i}p_{X}\left(x_{i}\right)\log p_{X}\left(x_{i}\right)$$

and is a measure of the uncertainty about the value that the random variable will take. If we are dealing with a random process, then the per sample entropy associated with the random variables $X_{1},X_{2},\ldots X_{n}$ which are samples of the random process given by

$$H_{n}=-\frac{1}{n}\sum p_{X_{1}X_{2}\ldots X_{n}}\left(x_{1},x_{2},\ldots ,x_{n}\right)\log p_{X_{1},X_{2},\ldots ,X_{n}}\left(x_{1},x_{2},\ldots ,x_{n}\right).$$

Shannon [21] showed that for a stationary source the limit of $H_{n}$ will converge to the entropy of the source *H*:

$$H=\lim_{n\to \infty }H_{n}.$$

Given two random variables *X* and *Y*, we can also define a *conditional entropy*

$$H\left(X|Y\right)=-\sum_{x,y}p_{XY}\left(x,y\right)\log p_{X|Y}\left(x|y\right).$$

This quantity can be seen as the uncertainty that remains about the value *X* will take on when the value of *Y* is known. If we take the difference between H(X), the uncertainty about *X*, and H(X|Y) the uncertainty that remains about *X* when *Y* is known, this difference is simply the information about *X* contained in *Y*. This quantity is called the *average mutual information* and is denoted by I(X;Y),

I(X;Y)=H(X)−H(X|Y).

We can show that the average mutual information is symmetric

I(X;Y)=I(Y;X).

If we consider *X* to represent the input to a noisy channel and *Y* the output of that channel, we can define the *capacity* of the channel as the maximum value of the average mutual information where the maximization is performed over the distribution of the input *X*.

## 3. Information Transfer over Neurons

From an objective point of view, the most reasonable place to apply information theory is the transmission of information over neurons, as, in this case, we can postulate a standard communication paradigm. The channel input is the stimulus, the channel itself is the neuron, and the channel output is the response. We divide our review into two parts. We first look at studies of single neurons, looking at possible ways that information is coded in neurons, and their capacity. We then move to multi-neuronal signaling and population coding.

### 3.1. The Single Neuron

Neurons communicate through trains of action potentials or neuronal spikes; therefore, these spikes, or combinations of them, can be viewed as the symbols of information-bearing codes traversing the channel. For this paradigm to have meaning, the neurons selected for the experiment have to be responsive to the type of stimulus selected. For example, to understand the behavior of auditory neurons, the neuronal activity of auditory nerve fibers was monitored when responding to an auditory stimulus [22]. In a different study [23], visual stimuli were provided to a trained rhesus monkey and the neuronal activity of a directionally selective neuron was recorded along with the psychophysical response of the rhesus monkey. In each case, the type of neuron was matched to the specific type of stimulus and response for which it was designed. At first glance, it seems that not only should the input be appropriate for the type of neuron, but the properties of the input should be tuned in the sense that the neuronal activity is highest for the tuned stimulus—the frequency at which the response is highest for an auditory nerve fiber, or the particular orientation of targets when testing optical neurons. However, Butts and Goldman [24], using a measure based on the average mutual information, and using experimental evidence, have shown that this is not always the case. When detecting stimuli in cases where the signal to noise ratio is high, stimuli close to each other are best discriminated not where the neuronal activity is highest but where the tuning curve—the plot of neuronal activity versus stimulus—has a high slope. In *Sense and the Single Neuron*, Parker and Newsome [25] provide a detailed view of the many experiments conducted to understand the behavior of single neurons. Regardless of the type of neuron, the neuronal response to the inputs is known to be noisy [26] dictating a bound on the amount of information that can be transferred over neurons [27], or, in other words, the capacity of the neuronal channel.

One can use a number of different approaches to estimate the capacity of the channel. One approach is to postulate a “coding” which makes use of the spikes. Both spike timing and spike frequency have been proposed as modes of information transfer, each resulting in a different estimate of the capacity of the neuronal channel. The spike frequency is proposed as the method of transmitting information that results in the extension of muscle fibers in [28]. By estimating the number of distinguishable frequencies and assuming that each of the distinguishable frequencies occurs with equal probability, the author obtains an estimate of the information that can be transferred over this particular neuronal channel. The maximum information transfer rate is estimated to be 12 bits/s. However, the author points out the lack of precision in this approach. A similar approach is used in [29] to estimate the capacity of mechanoreceptive cutaneous afferent fibers. In this case, the authors estimate the capacity of these fibers to be 3 bits/s.

If we treat neuronal signaling as a pulse code modulation scheme, then the smallest interval between two spikes provides an absolute limit on the amount of information that can be transferred. This interval would include the refractory period needed for the calcium channel to depolarize as well as the time for the signaling itself. A more efficient method would be pulse position modulation where the location of the pulse itself would contain the information. In the cognition literature, this kind of coding is referred to as spike interval coding. Here, the upper bound on the neuronal channel is determined by the smallest distinguishable interval. Based on the higher efficiency of this form of information transfer, MacKay and McCullough [30] proposed that this “ought” to be the modulation scheme used in the nervous system. Rapoport and Horvath [31] estimated that an upper bound on the capacity of such a scheme using biologically reasonable parameters would be about 4000 bits/s. This value is generally considered to be highly optimistic. Stein [32] points out this possible overestimation could be due to a large number of simplifying conditions. The stimulus was artificially generated for the nerve cells in a systematic fashion allowing for a larger number of spikes—perhaps an order of magnitude larger than would naturally occur. In practice, a stimulus does not always elicit a response and a response is sometimes generated when there is no stimulus. Stein [32] favors the idea of a frequency code where the information is encoded in the frequency of spikes.

Borst and Theunissen [33] use the relative independence of the response of neurons at different frequencies to propose a method for obtaining the capacity of a neuron. The same stimulus is presented a number of times to a neuron and the response recorded. For example, the stimulus may be a moving grating presented to a fruit fly and the response some function of the spike train recorded from a motion sensitive nerve cell. The responses are averaged to generate an average response and the difference between each response and the average is viewed as the neuronal noise. Looking at these signals in the frequency domain, at different frequencies, we can obtain the distribution of the response *R* and the distribution of the noise (the difference between individual responses and the average response) from which we can obtain their respective entropies. If we view the entropy of the neuronal noise as the conditional entropy of the response, given the stimulus, we can compute the average mutual information as

I(R;S)=H(R)−H(R|S).

By assuming the response at different frequencies to be independent, the total rate of information transfer can be obtained by adding the average mutual information at different frequencies.

In such experiments, both the input and the output can be statistical in nature which makes them a natural fit for information theory. If the stimulus has a statistical distribution, one can estimate the input entropy. The response is almost always statistical in nature. The distributions of the stimulus and response can be used to obtain an estimate of the average mutual information from which one can determine the capacity of the neuronal channel. Assuming a gamma distribution for the interspike interval, it has been shown [27] that the capacity achieving distribution in both the spike frequency and the spike interval coding scenarios is a discrete distribution. The capacity in this particular work is estimated to be around 15–50 bits per second.

Berger and Levy [34] come down firmly on the side of spike interval coding as the mode of information transfer, at least for cortical neurons. They model an idealized integrate and fire neuron in the sensory cortex as a multiaccess partially degraded broadcast channel. These cortical neurons have tens of thousands of afferent neurons that transfer their spikes, or action potentials, through dendrites to other neurons. Berger and Levy [34] use a communication model by viewing the computational activities of a neuron as a function of the spiking activities of its set of afferent neurons. The neuron then passes this computed information about the spiking activity of its set of afferent neurons to a set of efferent neurons. The authors define a random variable A(t) that is a function of the spiking propensity of the excitatory and inhibitory afferent neurons. They show that, under reasonable assumptions, A(t) is a random Poisson measure which can be approximated by a piecewise constant random process Γ(t) that has a constant value $\Gamma_{k}$ during the *k*th inter-spike interval. In their communication model, the authors use the random variable Γ as the input—recall that Γ is a function of the spiking activity of the afferent neurons—and the inter-spike interval *T* as the output. They define the rate of transfer as the average mutual information between Γ and *T* and show that the probability distribution function for the inter-spike interval *T* for an energy efficient neuron—one that maximizes bits per Joules—is a Δ-delayed gamma distribution, where Δ is the refractory interval of the neuron.

The authors present several arguments for selecting the interspike interval as the information-bearing signal including the fact that the information in the cortex is propagated through a sequence of neurons. In this case, using the frequency of spikes would increase the time required for information transfer beyond what is physiologically observed. Note, however, that the input in the authors’ communication model Γ is in a sense a function of the frequency of spikes in its afferent cohort. This model has been further developed in [35,36,37,38,39].

Evidence of the importance of spike timing can also be obtained by looking at how the time resolution used to analyze a spike train affects the computed information contained in it [40]. Reducing the time interval Δt will reduce the number of spikes per interval. If we look at the range of values in each interval Δt as the alphabet and estimate the information as the negative log of the alphabet size, we can obtain an information rate. As we decrease Δt, the value for which the information rate begins to drop is the temporal precision. Various experiments [40,41] have shown spike timing to be an important bearer of information.

So, do neurons use a frequency code or an interval code? While there is still considerable debate, the answer seems to be yes. For cortical neurons, the preponderance of opinion seems to be in favor of the interspike interval as the neural codes. Arguments for this include both the time sensitivity of these signals as well as the energy efficiency of this kind of coding. Frequency codes are exponentially more costly in terms of energy expenditure than interspike interval codes [42] and given the number of neurons in the cortex, if the choice is between a frequency code and an interval code, the advantage would seem to be with the interval code. Where the density of neurons is not as high and the stimulus tends to be slowly varying, a frequency code is a more robust alternative [43]. Given the promiscuity of biology, it is likely that organisms use both alternatives, as well as others, as the need arises.

### 3.2. Multiple Neurons and Population Codes

The response of a neuron to a stimulus can be highly variable—this variability allows us to apply information theoretic ideas developed for stochastic ensembles to the neuron, invoking the idea of multiple realizations of a random process. In the brain, we are not dealing with a single neuron but a large collection of neurons. The response of these neurons, in close proximity to each other, to a stimulus turns out to be highly correlated. If we look at each of these correlated responses as realizations of the same stochastic process, it would be reasonable to assign the difference to some kind of “noise” and average the responses. However, it turns out that such averaging results in a loss of information [44,45]. Experimental measurements in the primate motor cortex have shown that the direction of motion can be predicted by the weighted vector sum of the directions predicted from individual neurons where the weights correspond to the strength of the response [46,47,48]. The exact trajectory of the eye during saccadic eye movements is controlled by a bunch of collicular neurons. Lee et al. [49] have shown that the precise saccadic movement is a result of the weighted average of the activity of the individual neurons.

One of the difficulties with understanding the behavior of these neurons is in understanding the nature and level of interdependence. Ince et al. [50] provide an information theoretic approach to answering this question by looking at the average mutual information between stimulus and response for groups of neurons. The fundamental equations used are the same as in [33]—compute H(R) and H(R|S) and obtain the average mutual information as the difference. However, when dealing with bundles of neurons, the possible numbers of inputs and responses is very high. Consider a system of *C* neurons with a maximum of *M* spikes per neuron; then, the possible size of the input alphabet is ${\left(M+1\right)}^{C}$. Given this large size, empirically estimating the various probability density functions needed to obtain the entropy and conditional entropy can be problematic resulting in significant biases. Ince et al. [50] describe various approaches to overcoming the experimental limitations and then apply their methods to the particular task of obtaining the information transfer over cortical neuron corresponding to the whiskers of anesthetized rats. The stimulus set was different sinusoidal vibrations characterized by whisker velocity. They noted that the responses for different neurons were correlated in a stimulus-dependent fashion. To understand the level of interaction between neurons, they obtained the average mutual information by using the average of single neurons, pairs of neurons, triplets of neurons, etc. These were then compared to the estimated average mutual information for the population. They found that, while the effect of interaction increases significantly with population size, using pairs of neurons provides a reasonable estimate of the information transfer rate. Thus, the population structure can be captured by looking at the pairwise interactions between neurons.

These and other [51,52] efforts have shown that the use of information theory can help understand the question of dependency and redundancy in receptive fields and the population structure in multi-neuronal communications.

## 4. Entropy and Information in Cognitive Tasks

Neurons and collections of neurons can be modeled as channels of information, the stimulus and response can be measured experimentally and modeled statistically, and information theoretic concepts can be applied relatively easily. However, when we turn to the more subjective aspects of cognition, the situation becomes slightly more complex. While we can generally categorize and model the stimulus, it can be difficult to observe the response. Instead, we often have to make do with a proxy for the response. Furthermore, understanding what the channel is, let alone modeling it, can be a very difficult task. Despite these difficulties, there have been a number of interesting and ingenious applications of information theory to the process of cognition. We will begin with the simple case—selection from a random assortment of items—and then proceed to the notion of cognitive models and predictive coding in cognition. At each level, we will look at both the theoretical and empirical evidence for the validity of the use of information theory to analyze and understand cognitive processes.

### 4.1. Hick–Hyman Law

The fact that the entropy of equally likely outcomes from a set of *M* possible outcomes is log(M) is well known in information theory. Hick’s law [53] is an application of this to choice experiments where the subject responds to a set of equally likely stimuli. The original data for Hicks work comes from the work of a German philosopher Merkel in 1885 who conducted a number of experiments measuring the response time to a multiple choice task. The stimulus consisted of Arabic numerals 1 through 5 and the corresponding Roman numerals I through V. Each number was associated with a finger—the Arabic numerals with the fingers of the right hand and the Roman numerals with the fingers of the left hand. The subject waited with fingers on ten keys and raised the finger corresponding to the stimulus. Merkel was attempting to divide the time an individual took to respond to a stimulus between time required for cognition and time required for choice. Hicks [53] and later Hyman [54] studied the response time as a function of the number of choices and noted that the response time varied logarithmically with the number of choices. Hicks treated no stimulus as a possible choice for which the reaction time was zero and came up with a response time of αlog(M+1) where α was slightly different for Merkel’s data and for his data. Notice it is log(M+1) not logM because no stimulus is treated as one of the possibilities.

Hyman [54] repeated Hick’s experiments with a different experimental setup. He used a 4×4 array of lights with the stimulus being the lights at the four outer corners and the four lights forming the inner square. The subjects responded by speaking out. When he presented the stimuli with equal probability, he got the same results as Hick and Merkel—the response time varied with the log of the number of alternatives. Then, he extended the experiment to stimuli with unequal probability. He found again that the response time was correlated to the entropy of the experiment. He also found that the self information [55]$\log\frac{1}{p_{i}}$ of the *i*th experiment did not correlate with the response time. Recall that in each of these experiments the subjects were trained and therefore had a feel for the statistics of the stimulus. The response times were related to the average self information—or entropy—not the self information. Hyman’s result is much stronger evidence that entropy is a measure of information from a neural perspective rather than simply a statistical measure useful only for coding theory. Hick’s results simply showed that the response time varies as the log of the number of choices available. The fact that the log of the number of equiprobable choices is also the entropy might simply be a coincidence. Hyman’s results do not allow for such easy dismissal.

The Hick–Hyman effect has been replicated in a number of settings. Simpson and Huron [56] have shown that the reaction times when identifying pitch [57] follow the Hick–Hyman law.

With the recent development of tools to visualize the brain, the Hick–Hyman experiments have been conducted with the brain of the being monitored using fMRI [58]. The results show that the mediator for the response in the Hick–Hyman experiments is the set of regions in the brain that make up the cognitive control network. This is a set of six cortical regions—anterior cingulate cortex/pre-supplementary motor area, dorsolateral prefrontal cortex, inferior frontal junction, anterior insular cortex, dorsal pre-motor cortex, and posterior parietal cortex [59]. We will see some of these regions again when we discuss cognitive control. Wu et al. [58] confirm the association between the level of activation of the cognitive control network and the entropy of the task.

How has cognition in a more universal sense been studied using information theory? There are a number of approaches, the two more popular being cognitive control and model based cognition or predictive coding.

### 4.2. Cognitive Control

**Figure 1:**
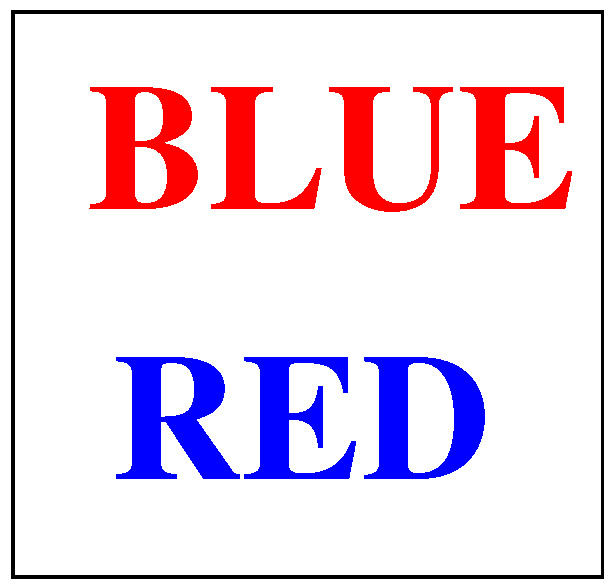
Stimuli for the Stroop test.

Cognitive control refers to a dynamic resource allocation strategy used by the brain to allocate cognitive resources to information relevant to the task at hand. A commonly used simple example where there is a need for resource allocation is the Stroop test [60] where the name of one color is written in another color. For example, the word “blue” may be printed in red type or the word “red” may be printed in blue type as in Figure 1 (in his original experiment, Stroop used the colors red, blue, green, brown, and purple). The subject can be asked to name the color ignoring the meaning of the text. Stroop found that, when the color was incongruent with the word, the response time increased by 74%. The increase in response time is due to the uncertainty or conflict.

There is also an auditory version of the Stroop test [61] where the subject is asked to judge whether an auditory input is high or low pitched, loud or soft, and fast or slow.

We are no longer dealing here with simple stimulus response, but issues of selective attention and control. This is a far more difficult situation to model than the situation in the Hick–Hyman effect. Berlyne [62] provides an entry point by trying to develop a measure of conflict. In a manner similar to Shannon in his landmark work [21], Berlyne develops the requirements for a degree-of-conflict function and finds that while entropy fulfills most of these requirements, it does not take into account the absolute strength of competing responses. Koechlin and Summerfield [63] take a different and very ingenious approach to the problem of cognitive control. In their development, they use particular realizations of stimulus and action. Here, we will use the more common approach of looking at the interactions between random variables. Let *S* be the random variable taking its value for the set of possible stimuli and *A* be the random variable which takes its values from the set of action. Then, the average mutual information between the stimuli and the actions is given by

I(S;A)=H(A)−H(A|S),

which can be rewritten as

H(A)=I(S;A)+H(A|S).

If H(A) is the average amount of information required for selecting an action in response to a stimulus, then it consists of two distinct terms: the average mutual information I(S;A) between the stimulus and action and the conditional entropy H(A|S). Koechlin and Summerfield interpret this latter term as the information required to take an action that is not contained in the stimulus. They call this term the information associated with cognitive control. They refer to the mutual information as the “sensorimotor control.” This formulation allows them to incorporate multiple levels of context. Let *C* be a random variable corresponding to a set of contexts. Then, we can define the conditional average mutual information between the context and action given the stimulus as

I(C;A|S)=H(A|S)−H(A|S,C)

or

H(A|S)=I(C;A|S)+H(A|S,C).

Thus, the average amount of information needed to take action, given a stimulus and a context for that stimulus, is given by a sum of three terms: (1) the average mutual information between the stimulus and action, (2) the average mutual information between the context and action given the stimulus and (3) the uncertainty about the action even with the knowledge of both the context and the stimulus:

H(A)=I(S;A)+I(C;A|S)+H(A|S,C).

This last term which depends on neither the context nor the stimulus could be the information related to past events. Koechlin and Summerfield call this episodic control. Using a set of forced response color letter tasks with brain regions monitored using fMRI Koechlin et al. [64] have shown that each of these terms is associated with distinct regions of the prefrontal cortex. The tasks are manipulated to activate different kinds of controls. Activity in the anterior portion of the dorsolateral prefrontal cortex varied with the amount of episodic information H(A|S,C), activity in the posterior dorsolateral prefrontal cortex varied with H(A|S) or the sum of the episodic control information and the contextual information and the activity in the premotor cortex varied with H(A). The organization of these areas is along an axis from the anterior to posterior of the lateral prefrontal cortex. This particular hierarchical organization of the brain in cognitive control tasks has been validated by other studies [65,66,67,68,69] as well.

These results are for a restrictive set of tasks and cognitive control in general probably involves much more than these particular regions [70,71,72]. The striking aspect of this work is the ability to use information theoretic concepts to understand the hierarchical and partitioned nature of cognitive control. Observing gambling tasks, Daw et al. [73] have found these regions to be involved in the exploratory as opposed to the exploitative tasks.

One area in particular that is often implicated in cognitive control is the anterior cingulate cortex [58,74,75,76,77], which seems to be a hub of a small world network dealing with cognitive control and shows an increase in activity with uncertainty. While the issue of where cognitive control resides in the human brain is far from settled, information theory can be useful in the careful design of experiments to understand the processing of information.

## 5. Model-Based Cognition

Let us step back now and take a broader look at cognition. If we examine the various cognitive tasks with which we are faced, we recognize a continuity. Unlike some of the tasks we have been talking about we are not faced with a fixed set of inputs. In *Whatever next? Predictive brains, situated agents, and the future of cognitive science*, Andy Clark [78] points out that it seems that the brain has an impossible task. Enclosed within the skull and with indirect access to the world through neuronal spikes, it has to make sense of its inputs. The only feasible way it can handle the continuing deluge through its senses is to establish a model, or models of reality. This idea of a cognitive model of reality has a long history. It exists in the notion of *umwelt* coined by the Estonian philosopher Jacob von Uexküll (1864–1944) as described by Kull [79]. *Umwelt* is a model of the world and Uexküll thought that the brain contains the world, or at least a model of it. Uexküll considered all living things to have their own *umwelt*, but, for the moment, we will concern ourselves mostly with humans. If we consider the cognitive task as the updating of the cognitive model, we can try and see if the information cost and the cognitive costs are related. Similar to the idea of predictive coding [80,81], the information contained in the update is more expensive when the update does not correspond with the model than when it does. Such predictive coding models have become a popular setting for understanding cognitive function [78,82] or perhaps even consciousness [83]. In his classic work, Andy Clark [78] traces the idea that the brain is a prediction machine from Helmholtz treatise on physiological optics in 1860 to the current day. Gregory [84] proposes that perceptual errors in the form of illusions be used to discover the underlying models, or, as he puts it, hypothesis. Piaget’s theory of cognitive development seems to embrace a predictive model [85]. Children develop a model of the world, observe the prediction error and adapt the model. *Figurative intelligence* deals with the static aspects of reality—the model, while *Operative intelligence* deals with the dynamic aspects of reality—prediction error and model update—or *accommodation*.

We begin this section with a look at visual cognition, one of the better understood cognitive functions. We will not invoke information theory much—the goal being simply to emphasize the fundamental importance of cognitive models and the important role of prediction. We will continue with a more general look at the use of cognitive models and delve into various applications of information theory which support the idea that many of the mechanisms of cognition can be understood from an information theory perspective.

### 5.1. Perceptual Information Processing Involves Processing Prediction Errors

One of the fundamental insights of Claude Shannon was that information is contained in events that are a departure from the norm. In order to measure the departure from the norm, one has to have a model of the norm. Events that do not conform to the model contain information—the amount of information depending on the deviation from the model. Any system that processes information will attempt to optimize the use of its resources by creating a model of the information generation process, and then focus its resources on those aspects of the signal that deviate from this model. The brain seems to be no exception to this. There is now increasing evidence that auditory and visual perception among others follows this paradigm [78,86,87,88,89].

**Figure 2:**
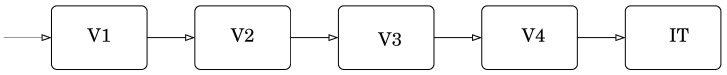
Hierarchical processing for object recognition.

**Figure 3:**
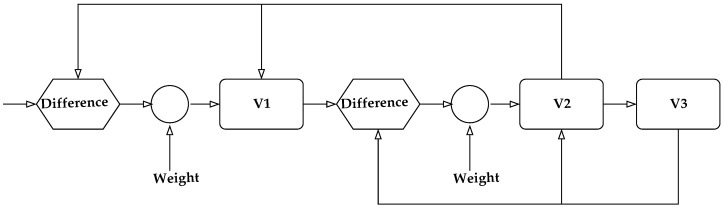
A prediction error model of visual perception.

Consider the visual recognition and identification of objects. Visual perception was assumed to work in a hierarchical manner with increasing abstraction as the signals progressed through the various visual areas in the brain [90,91], including the primary visual cortex (V1), prestriate cortex (V2), third visual complex (V3), visual area V4, and the inferior temporal cortex (IT). The primary visual cortex contains a large number of neurons containing a map of the visual field. The retinal image gets projected on the cortex in the visual area V1. The neurons in this area respond selectively to stimuli of differing orientation, spatial and temporal frequency, and color. Thus, V1 neurons provide the basic primitives that make up an object. These basic pieces are organized into increasingly more complex objects as the information is conveyed through V2 and V4 to the inferior temporal cortex. A simplified block diagram of the process of information flow is shown in Figure 2. Thus, if we view a box, the information about the edges is processed in the V1 region, which is passed on to the V2 region which organizes the edges and passes them on to the V4 region where perhaps the information is consolidated allowing the IT region to decide that the object is a box. Now, suppose we move our head. The box is now in a different part of the visual field, exciting different neurons in the V1 region, which then passes the information on to the V2 region and so on. This is tremendously wasteful as there are a large number of objects in our visual field and most of us tend to move our heads relatively often. If each time the information processing has to start from scratch—edges to planes to box—the amount of resources devoted to just image processing would have to be extraordinarily high. The same would be true for auditory and other sensory inputs. The current thinking [86], supported by empirical evidence is that each layer in the visual cortex models and predicts the input to the previous layer, and the information that gets passed on is the difference between the prediction and the input. The model construction has both a long-term and a short-term component with input from many parts of the brain. A possible simplified diagram of the information flow is shown in Figure 3. This looks more complicated than Figure 2; however, this complexity allows for a significant reduction in the need for information processing. Information from other parts of the brain and the state of each level is used to generate a prediction of the possible inputs to a lower level. Information flow from the lower level to the upper level takes place only when the prediction based on the model at the upper level is different from the input to the lower level. Therefore, if the model in the upper level is accurate, no information flows into the upper level obviating the need for processing. If on the other hand the model is inaccurate, information about the error in prediction (commonly known as prediction error) flows from the lower level to the upper level, inducing an updating of the model. As the world, in general, does not change in surprising ways, this considerably reduces the amount of information that has to be processed as we go merrily turning our heads to view objects. Each time we turn our head, the information about the motion of the head and the previous location of the box would allow for quite accurate predictions about the composition of the visual field, resulting in very little stimulus proceeding up the forward path. In our block diagram in Figure 3, we have also included a “weight” input. This in some sense represents the attention we are paying to our perception. If there is very little attention being paid, the weight given to the prediction error will be small, resulting in an acceptance of the model even when it does not quite accord with the perceptual input.

Before perceiving anything, our brain constructs a model of reality. Given the richness of the sensual world, this model has to be probabilistic. Sitting at an outdoor cafe, when we look down the street, there is a probability that we will see a car, and there is a probability that we will not. If we see a car, there is a probability that it will be red, blue, or brown; that it will be a sedan, a truck, an SUV, and so on. The model for what we see when we look up is probabilistic. The form of the probabilities can change depending on the context. If we were to get up to cross the street, the model may become binary; is there a vehicle coming or not? As we cross the street, after ascertaining that the street is empty, the weight on the prediction error is bound to be high; any deviation from the model has to be immediately processed.

**Figure 4:**
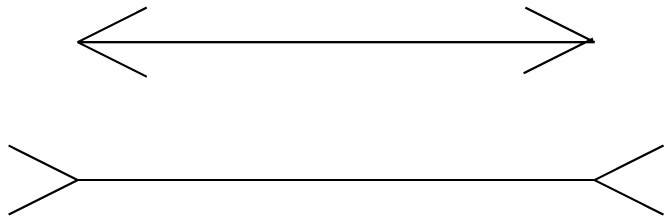
Muller–Lyer illusion.

There is considerable anatomical evidence for feedback connections from the higher to the lower levels of the perceptual hierarchy [86,92,93]. However, we can also see the effect of this paradigm without cutting into the brain. The overwhelming importance of the models our brains construct can be seen when we look at optical illusions. Consider the Muller–Lyer illusion shown in Figure 4.

Most people reading this will see the straight line on the bottom with two arrow tails as longer than the straight line on the top with two arrowheads even though they are actually the same length. The depth of the illusion can be quantified by increasing the length of the line on the top until the two lines appear the same length. Clearly, such a widespread illusion about something so fundamental as the length of a line must be the result of some structural feature of the perceptual mechanism—turns out it isn’t. The illusion doesn’t seem to work in nomadic societies. There are several possible explanations for this [94]. One is the “carpentered environment” hypothesis which says that we are so used to seeing edges that the arrow heads, or arrow tails on the straight lines make the lines seem like a part of a three-dimensional structure. In such a structure, the line on the top looks like an edge of a building with two walls going away from us while the line on the right looks like a corner with walls coming toward us. The impression is then that the line on the bottom is farther away and, therefore, it must be longer than the line on the left to appear the same length. Another hypothesis is that we are so used to seeing drawings of three-dimensional objects in two dimensions that we impute distances, and hence, using the same line of reasoning as above, we see the line on the bottom as longer. Regardless of which hypothesis we pick, what this tells us is that the world in which we live provides us with a model and the “objective” input of our senses is interpreted in terms of the model. If you grow up in a carpentered world, you have one model, and if you grow up in a nomadic environment far from structures with sharp edges you have a different model.

Consider another very different experiment: binocular rivalry [95,96,97,98]. In this experiment, the subject is placed such that each eye looks into a different mirror. In this way, different images can be placed in the left and the right eye. For example, a house can show up in one of the mirrors and a face in the other. If we look at the V1 region, both the neurons that would be triggered by a house image and the neurons that would be triggered by the face image show excitation. One would think that what the subject should perceive would be a blending of the house and face image. However, our life experience has taught us that what is viewed by the left and right eyes are very similar. Thus, what the subject ends up reporting seeing is either the house or the face for a period of time after which the perception switches, from house to face, or face to house [98]. When this experiment is performed with monitoring of the neural pathways, the forward information traffic immediately prior to the switch in perception is seen to be very high. The forward information traffic being the difference between the model and the perceptual input, this makes sense. The model in the upper level might say house, but the input from one eye does not agree with the predictions based on this model and we get a large prediction error. The upper level processes this large signal and after some time switches the model to that of a face, which in turn causes an increase in prediction error of a different sort.

One aspect these illusions emphasize is that our perception of the world is indirect. The brain comprehends the world through sensory inputs that are not, for the most part, a straightforward neural map. In order for the brain to make sense of the world, it has to construct a narrative consisting of a vast network of models—reason enough for using up 20% of the metabolic resources available to the human body.

We can understand how cognitive models use up metabolic resources by looking at the cognitive load associated with the updating of the model, which is the subject of the next section.

### 5.2. Cognitive Load

Neuronal activity incurs substantial metabolic cost [99,100]. Updating of cognitive models require significant neuronal activity and are, therefore, costly. Tasks that correspond to the encoding of large amounts of information (relative to a model) also have correspondingly higher cognitive costs. Zenon et al. [101] develop a formulation which connects cognitive cost to information cost. Their formulation makes insightful use of the Kullback–Leibler divergence to come up with an expression for the various costs incurred during a model update in terms of the average mutual information between the stimulus and model, the model and response, and the diversionary context (as in the case of the Stroop task) and the response. We begin with the definition of *surprise* associated with the observation of some data *D*. Itti and Baldi [102,103] define this surprise as the Kullback–Leibler divergence between the prior probability of a model *u* and its posterior probability given the observation or stimulus *x*. Let p(u) be the probability distribution of *u* prior to the stimulus *x* and p(u|x) be the distribution after the occurrence of the stimulus, then the surprise is given by

$$S\left(x,U\right)=KL\left(P\left(U|x\right)||P\left(U\right)\right)=\sum_{u}p\left(u|x\right)\log\frac{p(u|x)}{p(u)}.$$

If the base of the logarithm is 2, Itti and Baldi [102] call the unit of surprise *wow*. The idea is simple. If the observation comports with our cognitive models, there will be no difference between the prior and posterior models and hence no surprise. If the data does not comport with our model, there will be surprise, with the level of surprise depending on the divergence between the prior and posterior probabilities.

Zenon et al. [101] see this surprise as a means of computing the cost for updating the model. Taking the expectation of the expression for surprise with respect to *x*, we get

$$\begin{array}{ccc} E\left[KL(p(u|x)||p(u))\right] & = & \sum_{x}p\left(x\right)\sum_{u}p\left(u|x\right)\log\frac{p(u|x)}{p(u)}\\ & = & \sum_{x}\sum_{u}p\left(x\right)p\left(u|x\right)\log\frac{p(u|x)}{p(u)}\\ & = & I(U;X). \end{array}$$

This becomes informative when we consider that the average mutual information can be seen as the reduction in the uncertainty about the model after observing the stimulus:

I(U;X)=H(U)−H(U|X).

If the stimulus does not result in a significant update of the model, the cost is low, and, if the stimulus results in a significant update of the model, the cost is high. Thus, the cost of performing an unfamiliar task for which we do not have a good model would result in a high divergence between the prior and posterior distribution and, therefore, a high cognitive cost. This has consequences for behavior—we tend to avoid unfamiliar tasks and are comfortable performing familiar ones. In a similar manner, Zenon et al., obtain the cost of the response *y* by looking at the Kullback–Leibler divergence between the prior and posterior distributions of *y*

$$KL\left(p\left(y|u\right)||p\left(y\right)\right)=\sum_{y}p\left(y|u\right)\log\frac{p(y|u)}{p(y)}.$$

Taking the expectation of this divergence over the possible responses, we get the cost of the response as the average mutual information between the random variables corresponding model and the response, I(Y;U). Finally, for situations like the Stroop test where the stimulus conflicts with the task context, the authors model the increased cognitive cost as the Kullback–Leibler divergence between the automatic response p(y|u) and the response once the task context *t* is taken into account, p(y|u,t). Taking the expected value, we get the additional cost as the average mutual information between the task and the response. Thus, the total cost becomes

I(U;X)+I(Y;U)+I(Y;T|U).

With training, the statistical task structure of the unknown task becomes more familiar, leading to a decrease in the Kullback–Leibler divergence and hence the information cost. There is substantial experimental support for this assertion. Monkeys trained in responding to stimulus by eye movements showed increased activity in the supplementary eye field [104]—the region in the dorsal medial frontal lobe of the primate cerebral cortex involved in the control of saccades—when unfamiliar stimuli were initially presented. The level of activity died down as the monkeys got trained with that stimulus.

The cognitive load of task switching [105] can also be explained by this model. The individual initially learns the statistical structure of the first task. When task switching occurs the statistical structure changes and the increased cost is reflected in the Kullback–Leibler distance between the statistical structures. As might be expected, given this formulation, training reduces the metabolic cost [106]. The increase in the amount of information that it is necessary to process affects the desire of subjects for particular rewards. In App et al. [107], the authors used shifts of attention to increase cognitive load and showed that the increase in cognitive load leads to a discounting of the reward by the subjects. Foley et al. [108] in a study of how the brain guides active sensing, show that, given a choice, monkeys will select information that reduces uncertainty with respect to a model in a task. Finally, consideration of cognitive loads may require us to rethink how we measure age-related cognitive decline. As we age, we accumulate more and more experiences and, consequently, a larger number of models. This, in turn, means that stimuli could require the updating of larger numbers of models, which would increase response time that is often used as a proxy for cognitive performance. Ramscar et al. [109] show that the poorer performance of older adults on linguistic cognitive tests may be a reflection of the increased vocabulary of the individuals rather than an indication of cognitive decline.

A caveat is in order here. Updating models can become inefficient if the model becomes so detailed that the cost of the model becomes prohibitive [110]. This is reflected in the *minimum description length* principle [111] in source coding where the cost to be minimized is not simply the resources required to represent the data with respect to the model but the total cost of representing both the model and the data with respect to the model. One can reasonably assume that a similar principle is in operation for cognitive models.

## 6. Conclusions

Information theory is finally beginning to come into its own in its use as a tool for studying cognition. Where it was once an awkward import, the concepts and methods of information theory are providing a rigorous framework in which to study many aspects of cognition. The study and modeling of neuronal communications are becoming increasingly sophisticated with increasing synergy between advancements in measurement techniques and modeling approaches. While cognition as a whole is too complex an area to be explained by a single set of tools—however powerful they may be—we see from this survey that information theory is providing both a framework and a formalism—“a set of symbols and a precise way of combining and transforming them without any consideration either of the meaning or the reference of the symbols” [112], for providing a coherent understanding of experimental results. The steadily developing technologies of brain imaging are permitting the study of the brain and its functions to move beyond just behavioral experiments. As the measurements of brain activity increase in both temporal and spatial resolution, the arguments of the 1950s against the suitability of using information theory to study the processes of the brain are becoming less and less valid. Cognition allows us to navigate the world. Any disruption of this ability can have painful consequences. There are some preliminary indications [113,114,115] that information theoretic formulations can help us understand how some of these disruptions take place. As we increase our understanding of the brain and its functioning, information theory is very likely to be one of the enabling tools.
